# Comparison of total laparoscopic hysterectomy and abdominal hysterectomy

**DOI:** 10.4274/tjod.47108

**Published:** 2014-12-15

**Authors:** Osman Balcı

**Affiliations:** 1 Necmettin Erbakan University Meram Faculty of Medicine, Department of Obstetrics and Gynecology, Konya, Turkey

**Keywords:** Total laparoscopic hysterectomy, total abdominal hysterectomy, complications

## Abstract

**Objective:**

The aim of this prospective study is to evaluate and compare to the outcomes of total laparoscopic hysterectomy (TLH) and total abdominal hysterectomy (TAH) who performed in our clinic.

**Materials and Methods:**

We performed surgical procedures at Necmettin Erbakan University Faculty of Medicine, Department of Obstetrics and Gynecology between January 2013 and April 2014. Forty patients who underwent TLH (group 1) compared to 40 patients who underwent TAH (group 2). The mean age of the cases, body mass index (BMI), duration of operation, the amount of blood loss, rates of complications and post operative hospital stay were compared for two groups.

**Results:**

There were no statistically significant differences between the two groups regarding age, body mass index (BMI), specimen weight, pre-operative hemoglobin (Hb) value and rates of the complications. The mean post-operative Hb value was significantly higher in group 1 than group 2 (11.5±0.8 gr/dl vs. 10.8±1.7, p=0.02). The mean time of operation was significantly longer in group 1 than in group 2 (105.4±22.9 minutes vs. 74±18, p<0.001). The mean duration of hospital stay was statistically shorter in group 1 compared to the group 2 (2.48±0.6 days vs. 4.88±1.2, p<0.001).

**Conclusion:**

Total laparoscopic hysterectomy is safe and feasible method for gynecological diseases. TLH may offer specific benefits for properly selected patients. Its advantages are lower peri-operative morbidity, improvement of quality of life, shorter hospital stay and faster return to activity.

## INTRODUCTION

Gynecological surgical laparoscopy started to be used by Palmer at the end of 1950s. While surgical procedures like adhesiolysis, cyst aspiration and ovarian biopsy were performed firstly, Reich et al. reported first laparoscopic assisted vaginal hysterectomy case in 1989^(1)^. Since then, when compared with abdominal hysterectomy, because of lower morbidity and faster healing period, laparoscopic hysterectomy started to be used progressively as an alternative of abdominal hysterectomy. But because necessity of comprehensive surgical education and equipment today still a lot of gynecologists prefer abdominal surgery. Aim of this prospective study is to evaluate and compare the results of total laparoscopic hysterectomy (TLH) and total abdominal hysterectomy (TAH) cases which were performed in our clinic.

## MATERIALS AND METHODS

Eighty patients who had hysterectomy operation for benign indications between January 2013 and April 2014 at Necmettin Erbakan University Meram Medical Faculty, Gynecology and Obstetrics Department are included in this study. According to which surgical procedure performed, patients are chosen consecutively and divided into two groups. While group 1 involve 40 patients who had TLH operation; group 2 involves 40 patients who had TAH operation. All of the patients had pre-operative endometrial biopsy.

All of the patients were operated by same surgeon and both of these two groups had same pre-operative preparation. All of the patients were hospitalized 1 day before operation. Twenty-five mg oral diazepam was given each patient 2 hours before operation for premedication and operations were performed under general anesthesia. All of the patients received antibiotic prophylaxis pre and postoperative and both of these two groups received suitable analgesics for pain control. TAH was performed pfannenstiel incision with classical technique which was described for benign indications^(2)^.

To the patients who had TLH operation, after trendelenburg position, cervical length was measured for uterine manipulation, RUMI^®^ II (CooperSurgical, Trumbull, CT) manipulator was placed in uterine cavity. This preparation part of operation took 10 minutes. During surgery 10 mm telescope and advanced bipolar energy modalities were used. Including umbilicus 3 laparoscopic trocar were inserted, firstly 10 mm trocar was inserted directly in 1 cm incision which was on subumbilical area. Laparoscope was placed in abdomen after 3-4 L CO_2_ insufflations into the abdominal cavity. Second and third incisions were made on avascular right and left lower abdomen and two 5 mm trocars were placed in abdomen. All relations of uterus and vaginal complex were cut by use of bipolar electrocautery same as classic laparoscopic hysterectomy. Uterus and ovaries were put out of abdomen from vagina, and if necessary intracorporeal myomectomy and morcellation was performed. With closure of vaginal cuff using laparoscopic intracorporeal approach, operation was finished. Vaginal cuff was sutured with number 0 vicryl (Ethicon, Somerville, NJ) separately and sutures were passed through right and left utero-sacral and cardinal ligaments. Operation timing was done between skin incision and last skin suture. Patients’ preoperative and postoperative first day hemoglobin value was registered. Foley catheter was removed on postoperative first day. All of the patients were called for gynecological examination on postoperative 40^th^ day.

Groups are compared in terms of mean age, body mass index (BMI), operation time, blood loss, complication rate and postoperative hospitalization time.

Data was registered as mean ± standard deviation and percentage. Data analysis was done with chi square and student-t tests of SPSS program. p<0.05 value was regarded as statistically significant.

## RESULTS

Mean age and body mass index (BMI) of both of these two groups were similar and there was no statistically significant difference. There was no difference between two groups about previous abdominal surgery. Operation indications were similar for both two groups and major indication was myoma uteri. Patients’ characteristics and operation indications were shown on Table 1.

Weight of surgical specimen, preoperative hemoglobin (Hb) value and complication rates was similar for both two groups and there was no statistically significant difference. Mean postoperative Hb value was higher in group 1 than group 2 and this was statistically significant (11.5±0.8 gr/dl-10.8±1.7, p=0.02). Mean operation time was longer in group 1 than group 2 and this was statistically significant (105.4±22.9 minutes-74.5±18.1, p<0.001). Mean hospitalization time was shorter for patients who undergone TLH (group 1) than patients who undergone TAH (group 2) and this was statistically significant (2.48±0.6 day-4.88±1.2, p<0.001). There was necessity of blood transfusion for one patient who had TLH and for two patients who had TAH, this was not statistically significant. Neither of groups had intraoperative complications, 1 patient who had TLH and 2 patients who had TAH had postoperative fever. There was no need for laparotomy about patients who had laparoscopy. Clinical results of patients are shown on Table 2.

Postoperative pathology results were reported; leiomyoma at 38 patients, serous cyst adenoma at 12 patients, adenomyosis at 10 patients, simple serous cyst and leiomyoma at 7 patients, endometrial polyp at 5 patients, endometrioma at 4 patients, non-atypical endometrial hyperplasia at 3 patients and atypical endometrial hyperplasia at 1 patient.

## DISCUSSION

Even though abdominal hysterectomy is the most frequented way of hysterectomy in the world, today we have a lot of techniques for hysterectomy. Uterus may be removed abdominal, vaginal or laparoscopic.

In a lot of studies which compare abdominal and laparoscopic hysterectomy, because of lower complication incidence and lower postoperative pain, less blood loss, shorter hospitalization period, shorter healing time and earlier turn back to daily activities laparoscopic hysterectomy is reported to have more advantages than abdominal hysterectomy^(3,4,5,6)^.

Because of laparoscopic surgery needs experience, while laparoscopic hysterectomy cases take a long time at the beginning, with progressive experience operation time is getting shorter. Anyway, studies report that laparoscopic hysterectomy takes longer operation time than abdominal hysterectomy. Olsson et al. and Härkki-Sirén et al. and Çelik et al. reported correspondingly that operation time for TLH is statistically significant longer than TAH^(7,8,9)^. But Seracchioli et al. reported no statistically significant difference about TLH and TAH operation time at their clinical trial which includes 122 women who have bigger uterus than 14^th^ gestational week^(10)^. Similarly, Ribeiro et al. found out operation time shorter for vaginal hysterectomy, found out no difference between TAH and TLH in their randomized prospective study which consisted of 60 patients and compared abdominal, vaginal and laparoscopic hysterectomy^(11)^. In our study operation time was longer for patients who had TLH than patients who had TAH and this was statistically significant.

In a lot of studies intraoperative and perioperative blood loss in laparoscopic hysterectomy was less than abdominal hysterectomy^(7,12,13,14)^. Raju and Auld, Çelik et al., Seracchioli et al. and Ribeiro et al. found out no statistically significant difference about blood loss between TLH and TAH^(5,9,10,11)^. In our study postoperative hemoglobin value was higher in TLH patients than TAH patients and this was statistically significant.

When compared with open surgery procedures, laparoscopic surgery probably because of less tissue trauma and less inflammatory response, is related with less postoperative pain and shorter healing period^(4,5,7,8,12,13,14,15,16)^. Also, studies show that postoperative hospitalization time is shorter in laparoscopy group^(7,8,9,14)^. Like other studies, in our study postoperative hospitalization period is statistically significant shorter in laparoscopy group.

Complication rate in laparoscopic hysterectomy is close to other surgical procedures. Olsson et al. couldn’t find out statistically difference between complication rates of TLH and TAH in their study which compared 71 TLH and 72 TAH cases and also Çelik et al. couldn’t find out statistically significant difference in their study which compared 47 TLH and 30 TAH cases^(7,9)^. In the meta analysis of randomized controlled trials which Johnson et al. reported, when compared with abdominal hysterectomy, laparoscopic hysterectomy cases have more urinary tract injuries, but there is no statistically significant difference about other visceral organ injuries^(17)^. Garry et al. reported in their eVALuate trial, in which 1380 women included, laparoscopic hysterectomy is related with more complications than abdominal hysterectomy^(18)^. In our study any introperative complication wasn’t seen in both TLH group and TAH group. But 1 patient from TLH group and 2 patients from TAH group had postoperative fever.

In conclusion, laparoscopic hysterectomy is a safe and suitable procedure for chosen patients. It affords patients advantages like less peri-operative morbidity, better life quality, shorter hospitalization time, and faster return to activity.

## Figures and Tables

**Table 1:**
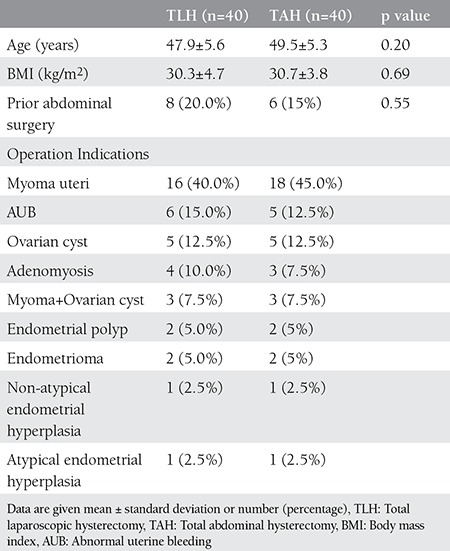
Patients’ characteristics and operation indications

**Table 2:**
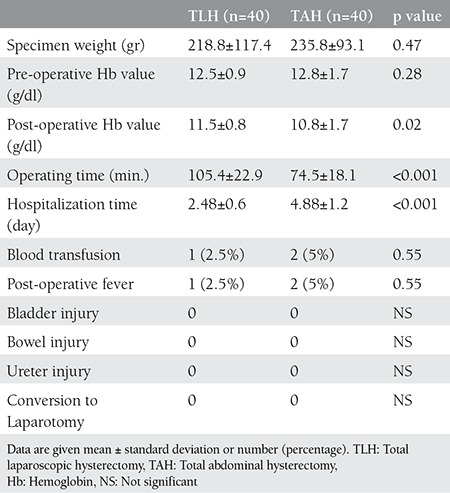
Clinical results of patients
